# FemXpress: Systematic Analysis of X Chromosome Inactivation Heterogeneity in Female Single‐Cell RNA‐Seq Samples

**DOI:** 10.1002/advs.202504754

**Published:** 2025-06-29

**Authors:** Xin Wang, Yingke Ma, Fan Li, Wentao Cui, Tianshi Pan, Siqi Wang, Sinan Ma, Qingtong Shan, Chao Liu, Yukai Wang, Ying Zhang, Yuanchun Zhou, Wei Li, Pengfei Wang, Qi Zhou, Guihai Feng

**Affiliations:** ^1^ State Key Laboratory of Stem Cell and Reproductive Biology Institute of Zoology Chinese Academy of Sciences Beijing 100101 China; ^2^ Medical School University of Chinese Academy of Sciences Beijing 100049 China; ^3^ Key Laboratory of Organ Regeneration and Reconstruction Chinese Academy of Sciences Beijing 100101 China; ^4^ Beijing Institute for Stem Cell and Regenerative Medicine Beijing 100101 China; ^5^ University of Chinese Academy of Sciences Beijing 100049 China; ^6^ National Genomics Data Center China National Center for Bioinformation Beijing 100101 China; ^7^ Beijing Institute of Genomics Chinese Academy of Sciences Beijing 100101 China; ^8^ Computer Network Information Center Chinese Academy of Sciences Beijing 100083 China; ^9^ College of Life Sciences Northeast Agricultural University Harbin 150030 China

**Keywords:** escape genes, scRNA‐Seq, X chromosome inactivation

## Abstract

X chromosome inactivation (XCI) is crucial for balancing X‐linked gene dosage in female cells by randomly silencing one X chromosome during early embryogenesis. However, accurately classifying cells based on the parental origin of the inactivated X chromosome in single‐cell samples remains challenging. Here we present FemXpress, a computational tool leveraging X‐linked single nucleotide polymorphisms (SNPs) to group cells based on the origin of the inactivated X chromosome in female single‐cell RNA sequencing (scRNA‐Seq) data. FemXpress performs robustly on both simulated and real datasets, without requiring parental genomic information, and can also identify genes that escape XCI. Applying FemXpress to single‐cell RNA‐Seq data from multiple tissues of a cynomolgus monkey, we reveal heterogeneity in XCI origin across organs and cell types. In each organ, we identify candidate XCI‐escaping genes, and within each cell type, we observe gene expression differences associated with XCI origin, potentially contributing to phenotypic variability. Furthermore, FemXpress demonstrated strong performance in phasing XCI in scRNA‐Seq datasets from embryos and colon tumors. In summary, FemXpress provides a powerful approach for XCI status analysis, offering new insights into XCI dynamics at single‐cell resolution.

## Graphical abstract

FemXpress is a computational tool that leverages X‐linked single nucleotide polymorphisms (SNPs) to group cells by the origin of the inactivated X chromosome in female single‐cell RNA sequencing (scRNA‐Seq) data. It demonstrates strong performance on both simulated and real datasets without requiring parental genomic information, and can also identify genes that escape X chromosome inactivation (XCI).

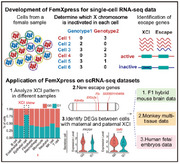

## 1 Introduction

In mammals, X chromosome inactivation (XCI) evolves into maintaining the gene dosage balance between XX females and XY males.^[^ ^1^ ^]^ During the blastocyst stage, each female cell randomly inactivates one of the two X chromosomes, and this inactivated status is stably inherited by all daughter cells.^[^ ^2^ ^]^ Generally, XCI is initiated by the long non‐coding RNA Xist, which recruits a series of silencing complexes, gradually leading to heterochromatinization of the Xist‐bound chromosome and ultimately resulting in the inactivation of the entire chromosome.^[^ ^3^ ^]^ There are also species‐specific mechanisms regulating XCI. For example, imprinting of the paternal X chromosome in cells that develop into an extraembryonic lineage is common in rodents, but whether this occurs in humans remains controversial. Additionally, certain histone modifications present on the inactivated X chromosome in humans are not observed in mice.^[^ ^4^ ^]^ Furthermore, XCI does not imply that the entire chromosome is transcriptionally silent. A subset of genes on the inactivated X chromosome can escape the silencing state and transition to an active expression pattern in a cell type‐specific as well as species‐specific manner.^[^ ^5^, ^6^ ^]^ As a result, different tissues of a female individual exhibit a mosaic pattern of XCI, in which the proportion of cells with inactivated X chromosomes of paternal or maternal origin is expected to be 1:1.

From the perspective of the origin of the inactivated X chromosome, heterogeneity exists in the inactivation of the paternal or maternal X chromosome, even among cells of the same cell type.^[^ ^1^ ^]^ Furthermore, cohort studies have revealed that XCI skew, which refers to a deviation from the expected 1:1 ratio, is enriched in aging and tumor populations.^[^ ^7^, ^8^, ^9^ ^]^ Therefore, unraveling the heterogeneity of the XCI status and accurately distinguishing the cell groups based on which X chromosome is inactivated in female cells is crucial for understanding the influence of differences in the parental X chromosome on various traits.

Traditional methods for detecting XCI heterogeneity typically involve polymerase chain reaction (PCR)‐based inference of the proportion of paternal and maternal X chromosomes inactivated in an individual by targeting allelic differences in sequence variants present in blood samples.^[^ ^8^, ^10^ ^]^ Alternatively, in model‐based research, the two X chromosomes can be labeled separately to distinguish their origin.^[^ ^11^ ^]^ However, the limitations and inaccuracies of these methods are apparent. For instance, PCR‐based approaches require prior knowledge of specific parental‐origin SNPs and the design of targeted primers. Additionally, such low‐throughput methods are unsuitable for detecting differences across various cell types. Similarly, strategies involving the separate labeling of X chromosomes are restricted to model organisms, limiting their applicability in broader contexts. Recently, bioinformatics tools such as scLinaX have been developed to analyze XCI heterogeneity at the single‐cell level. While scLinaX is able to quantify gene‐specific escape across different cell populations, it does not support the classification of cells based on the parental origin of the inactivated X chromosome.

In this study, we present FemXpress, an analytical tool for investigating XCI heterogeneity in female scRNA‐Seq samples, by clustering cells based on linked single nucleotide polymorphisms (SNPs) in expressed genes on the X chromosome. FemXpress achieved an accuracy of over 90% on cell classification for both simulated human data and real sequencing data from mouse. By identifying SNPs with biallelic expression in homogeneous XCI cells, FemXpress predicted potential XCI escape genes, among which several genes were previously reported. Subsequently, we applied FemXpress to multi‐tissue data from the same monkey. Our analysis revealed significant heterogeneity in XCI origin, not only between different cell types within the same organ but also across tissues. Additionally, we identified hundreds of XCI‐escaping genes across the three organs. Crucially, within the same cell type in each organ, we discovered genes that were differentially expressed because of differences in the parental origin of the inactivated X chromosome. These genes could potentially contribute to phenotypic diversity. Furthermore, we extended the application of FemXpress to additional public datasets, including single‐cell data from individuals at the same developmental stage and single‐cell data from colon tumors of different female patients.

Overall, FemXpress leveraged single‐cell data from female samples to unravel XCI heterogeneity and identify genes that escape XCI, providing a valuable approach for the precise analysis of female scRNA‐Seq samples, particularly in clinically relevant samples.

## 2 Results

### 2.1 Design Rationale for FemXpress

**Figure 1:**
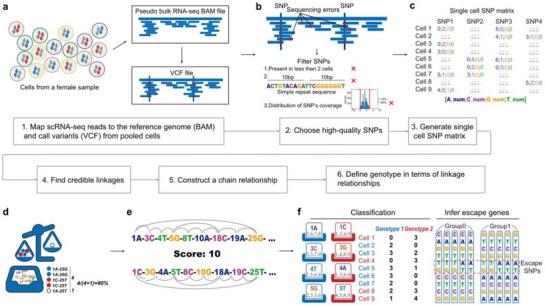
Graphical overview of the FemXpress method. a) Individual female scRNA‐Seq samples were aggregated as pseudo‐bulk for SNP calling on the X chromosome. Cells within a tissue are illustrated by circles in various colors, indicating different cell types. The RNA molecules shown in blue and red within these cells represent the expression of X chromosomes of different parental origin. VCF files were generated from the BAM files of pooled cells for genotype analysis. b) Stringent SNP filtering in pseudo‐bulk female scRNA‐Seq samples. Only loci that were genotyped in at least two cells were considered to ensure reliable variant detection. Secondly, SNPs found within simple repeat regions were discarded to avoid false positives commonly associated with sequencing errors in these areas. Lastly, the log ratios of reads for both genotypes at each position were calculated, and only the middle 80% of these ratios were retained to reduce the influence of extreme outliers. c) Genotyping data for identified potential SNPs were applied to subsequent analytical steps. d) Reliable linked genotypes were established between SNP pairs. e) Genotypes of linked SNP pairs were used to construct the longest parental‐origin X chromosome SNP linkage map. f) The X chromosome SNP linkage map was used to determine the XCI origin in each cell and to identify XCI‐escaping genes based on SNP genotype heterogeneity within cells sharing the same inactivation origin.

The workflow of the FemXpress tool is illustrated in **Figure** **1**. The primary objective of FemXpress is to partition female scRNA‐Seq samples into two distinct clusters based on the parental origin of the inactivated X chromosome. In addition, FemXpress aims to identify XCI‐escaping genes within each classified cluster based on the simultaneous expression of both alleles of a certain SNP. To achieve these goals, two crucial issues must be addressed: (1) identifying the SNPs that can accurately reflect the different genotypic characteristics between parental‐derived X chromosomes; (2) establishing reliable linkages of SNPs to either active or inactive X chromosomes.

SNPs demonstrating differences in parental origin were identified by aligning the scRNA‐Seq data to the reference genome as a pseudo‐bulk sample, thereby avoiding dependence on parental genomic information (Figure 1a). To minimize the impact of errors caused by the sequencing approach and analytic bias on SNP calling, strict filtering criteria were applied, including: (1) a minimum coverage of two reads supporting either genotypes of the SNP; (2) the detection of each genotype in at least two cells; (3) the exclusion of loci originating from simple repeat regions; (4) removal of top and bottom 10% SNPs based on reads ratio for each genotype at the position, since these are likely introduced by sequencing errors in high coverage regions (Figure 1b).

Individual SNPs are insufficient to classify the majority of cells due to the sparsity of gene detection in scRNA‐Seq methods, such as 10X Genomics. To address this limitation, we generated a high‐quality SNP matrix to specifically link chain SNPs to the different parental‐derived X chromosomes (Figure 1c). In general, establishing a link between two distant SNPs is challenging. However, in female somatic cell samples, only one X chromosome is stably inactivated. Therefore, if the genotypes of two SNPs are simultaneously identified in sequencing reads from at least one cell, these SNPs are located on the same active X chromosome. We counted the genotypes of the two SNPs that were simultaneously detected in each cell and defined them as linked genotypes only if both genotypes were present in at least 80% of the cells (Figure 1d).

Next, we conducted extended linkage scoring by combining the linked genotypes into longer chains of linked loci. The pair of haplotypes with the highest number of linked loci was selected as the genotype for the parental source X chromosome (Figure 1e). When the haplotypes of the parents were phased, the origin of the X chromosome for each cell was determined using a voting method. Each haplotype received one vote if the cell carried an allele base from that haplotype, and the cell was classified as having that haplotype if the votes were higher than those for other haplotypes. Finally, in cells already classified with the same parental source of the inactivated X chromosome, we examined the frequency of concurrent parental origin genotypes at each SNP position to identify potential XCI‐escaping gene‐derived SNPs (Figure 1f).

### 2.2 Performance of FemXpress on Simulated Data

**Figure 2:**
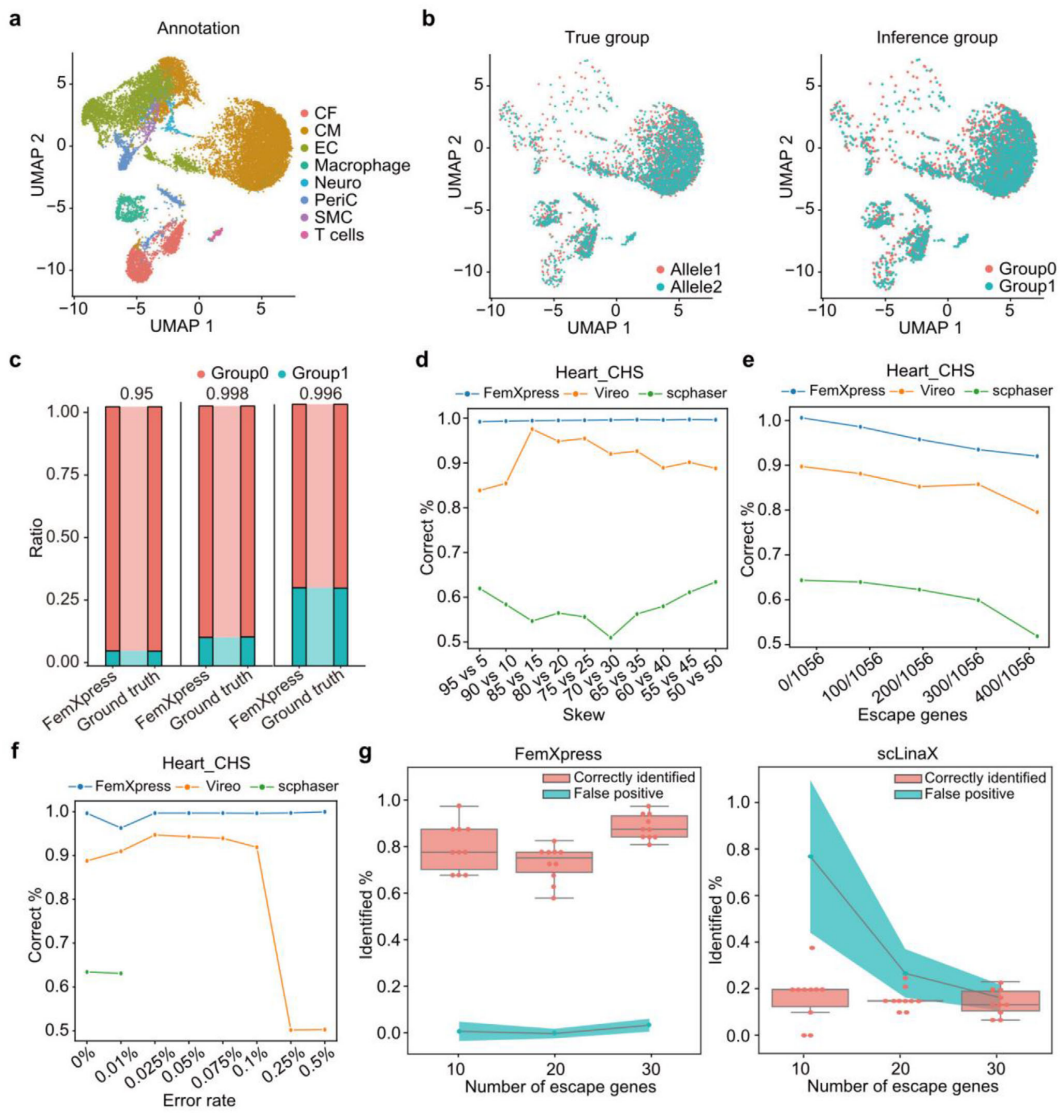
Evaluation of FemXpress using simulated data. a) UMAP embedding of human heart scRNA‐Seq data, with each cell color‐coded according to cluster identity and annotations denoting the respective cell types (CM, cardiomyocyte; CF, cardiac fibroblast; EC, endothelial cell; EpiL, epithelial‐like; PeriC, pericyte; Neuro, neuron; SMC, smooth muscle cell). b) The UMAP embedding shows human heart scRNA‐Seq data with cells color‐coded by XCI origin: the validated ground truth (left) and FemXpress's classification (right). c) Bar plot showing the agreement between FemXpress's classification and the established ground truth at an error rate of 0.05% for the XCI parental origin ratios of 95:5, 90:10, and 70:30, respectively. The prediction accuracy rates are annotated above each bar. d) The percentage of correctly clustered cells in simulated data generated with different levels of X‐skew (x‐axis), compared for FemXpress (blue), Vireo (orange), and scphaser (green). e) The percentage of correctly clustered cells in simulated data generated with varying numbers of full escape genes (x‐axis). In total, 1056 genes had read coverage greater than 100 in these simulations. Results for FemXpress (blue), Vireo (orange), and scphaser (green) are shown. f) The percentage of correctly clustered cells in simulated data generated with varying sequencing error rates (x‐axis) using FemXpress (blue), Vireo (orange), and scphaser (green). Due to scphaser's high memory usage, we obtained results only for the scenarios with no sequencing errors (approximately 63%) and with 0.01% error (approximately 63%) on a server with 512 GB memory. g) The correctly identified and false positive percentage of escape genes by FemXpress (left) and scLinaX (right) from 10, 20, and 30 genes randomly selected from the 55 genes which contain SNPs with read coverage > 100.

To evaluate the feasibility and performance of the FemXpress tool in identifying the parental origins of XCI, simulated data for XCI were generated using the public single‐nucleus RNA‐Seq dataset from female heart tissues (**Figure** **2**a)^[^ ^12^ ^]^ We mapped the scRNA‐Seq data to the human genome and focused on the alignment of the X chromosome. All mismatched positions were replaced with reference bases to obtain a perfect alignment of the X chromosomes. We then assigned a set of individual female SNPs obtained from 1000 Genomes trio family to these sequencing fragments.^[^ ^13^ ^]^ For each cell, we randomly inactivated one parent‐derived chromosome, meaning that the genotypes of all genes expressed on the X chromosome were derived from the other parental chromosome. Even on simulated data with an error rate of 0.05%, FemXpress achieved a cell classification accuracy of 99.7% (Figure 2b). Considering the presence of numerous samples with XCI skew, we performed sequencing simulations using different proportions of parental XCI. Even under highly imbalanced parental XCI proportions, such as 95:5, FemXpress exhibited stable performance (Figure 2c; Figure S1, Supporting Information). For the unprocessed dataset—without simulated sequencing errors, XCI skew, or other modifications—FemXpress completed the analysis in approximately 656 seconds on a server with 512 GB memory and 48 × 2.20‐GHz CPUs, using an input file of approximately403 MB.

Furthermore, we compared our tool with existing haplotype inference methods based on scRNA‐Seq data, such as scphaser^[^ ^14^ ^]^ and Vireo.^[^ ^15^ ^]^ These methods are designed for general chromosome phasing and do not leverage the unique feature of female cells, in which almost only one X chromosome is expressed. We evaluated these tools across a broad set of simulated scenarios featuring varying sequencing error rates, degrees of XCI skew, and numbers of X‐chromosome escapees. Since these two tools were not specifically designed for X chromosome phasing in female cells, FemXpress demonstrated consistently superior performance (Figure 2d–f). To demonstrate the stability of our method across different populations, we separately constructed simulated datasets using trio families from the CHS, CEU, and YRI populations, which vary in SNP density. The results showed that FemXpress performed well in clustering these simulated datasets across all three population samples (Figure S2a—f, Supporting Information). By progressively reducing the number of SNPs in the simulated dataset, we found that FemXpress maintained strong performance even when the SNP count was reduced to 1826 SNPs (Figure S2g, Supporting Information). To further evaluate FemXpress's ability to identify escapees, we tested it on simulated datasets with varying numbers of XCI escapees. The results demonstrated that FemXpress accurately detected escapees across multiple detection thresholds and outperformed scLinaX,^[^ ^16^ ^]^ a tool specifically designed to quantify XCI escape using scRNA‐Seq data (Figure 2g).

### 2.3 FemXpress Achieves Consistent Performance on Mouse Brain Cells

**Figure 3:**
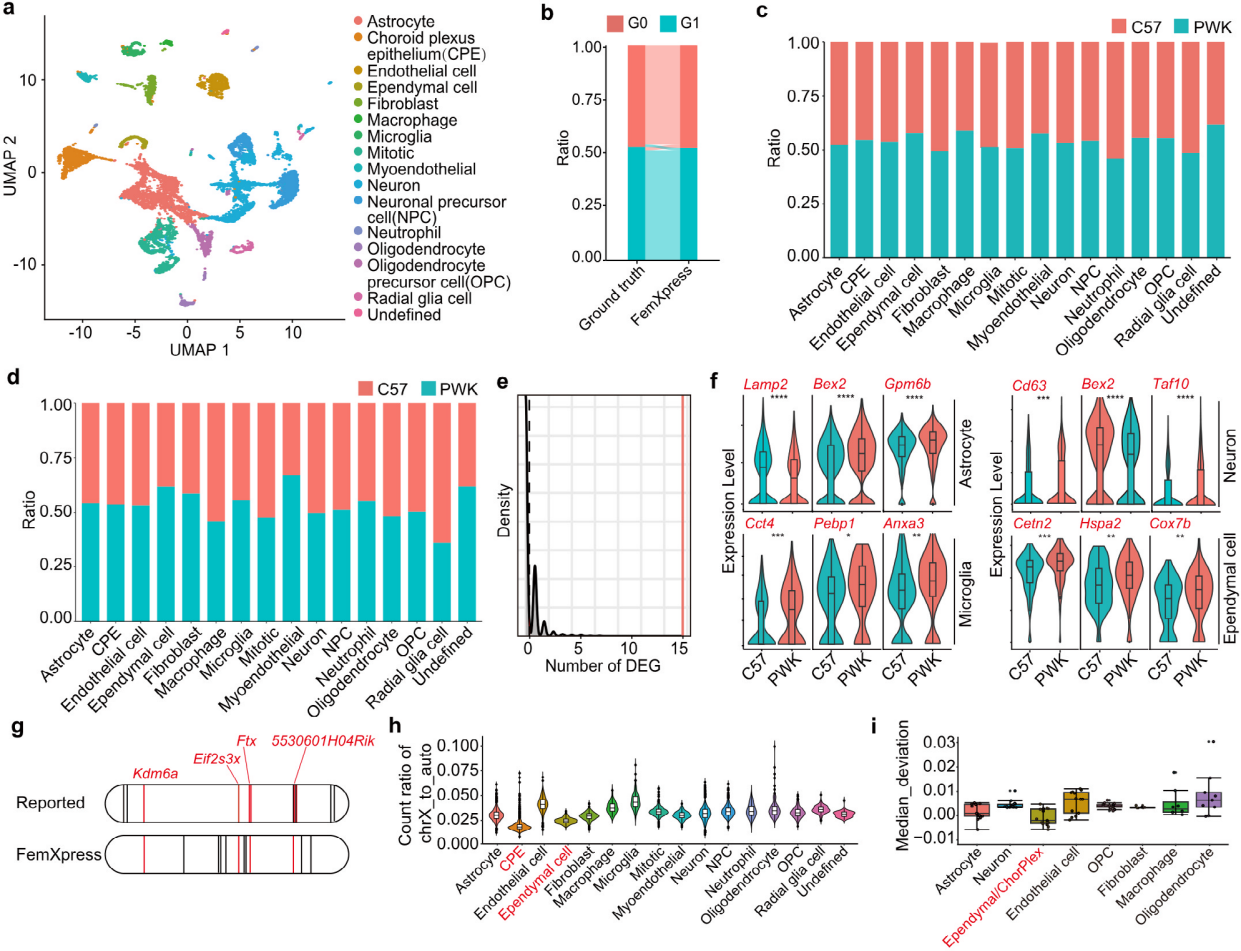
Assessment of FemXpress performance with mouse brain scRNA‐Seq data. a) UMAP embedding of mouse brain scRNA‐Seq data. Each cell is color‐coded according to cluster identity and with annotated cell types. b) Stacked histogram visualizing FemXpress classification of cell‐level XCI origins and the ground truth informed by parental genome data (G0: Group 0; G1: Group 1). c) Stacked histogram depicting cell XCI origin classifications within various cell types, according to parental genome data. d) Stacked histogram showing the distribution of cell XCI origin classifications within different cell types as predicted by FemXpress. e) Distribution plot showing the counts of differentially expressed genes (DEGs) from 1000 random splits of sample cells, with a red line indicating the DEG count from grouping by XCI origin. f) Violin plots of representative DEGs between cells with different parental XCI origin in specified cell types. g) Location distribution on the human X chromosome of potential XCI‐escaping genes in mouse brain, with those both reported and predicted by FemXpress highlighted in red. h) Violin plots illustrating the X‐to‐autosome read count ratios across different cell types in mouse brain tissue. i) Box plot displaying the distribution of X‐to‐autosome read count ratios for various cell types in mouse brain samples from a public database. The median X‐to‐autosome read count ratio was calculated across all samples, and the y‐axis represents the deviation of each sample's ratio from this median value. Negative values on the y‐axis indicate that a cell type has a lower X‐to‐autosome read count ratio compared to the median. Each point in the plot corresponds to a collection of cells of a particular type from an independent mouse brain sample.

To further evaluate the accuracy of FemXpress, we performed scRNA‐Seq on the brain tissue of female offspring resulting from crosses between C57BL/6NJ and PWk/PHJ strains. On a server with 512 GB memory and 48 × 2.20‐GHz CPUs, FemXpress completed inference in 48,352 s for 9859 cells (approximately1.8 GB) in the brain scRNA‐Seq data of the mouse. Based on the established cell type definitions, 15 cell types were annotated (**Figure** **3**a; Figure S3a, Supporting Information). Next, we performed genome sequencing of the parental X chromosome haplotypes to obtain a ground truth for further evaluation. FemXpress was able to precisely identify the source of XCI at both the whole tissue and cell‐type levels with single‐cell resolution without any additional genomic information (Figure 3b–d; Figure S3b,c, Supporting Information). To elucidate the impact of XCI from different parental sources on cellular function, we compared differentially expressed genes (DEGs) between cells with X chromosome inactivation from either parent source. Using the default parameters of Seurat,^[^ ^17^ ^]^ we identified 15 DEGs between the two groups, which significantly exceeded the number of DEGs discovered through random cell grouping (Figure 3e). Interestingly, some of these DEGs, which are regulated by the parental source of XCI for different cell types, have been reported to exhibit abnormal expression in the brain and may be associated with various brain diseases. For instance, genome‐wide analysis of epigenetic silencing identified *BEX1* and *BEX2* as candidate tumor suppressor genes in malignant glioma (Figure 3f).^[^ ^18^ ^]^ Moreover, FemXpress can predict and infer potential XCI‐escaping genes in which both alleles are concurrently expressed within a single cell. Therefore, we identified 12 potential XCI‐escaping genes in the brains of newborn mice. Although the repertoire of escaping genes varies across tissue types and disease states, we also found that four of them had already been reported (Figure 3g)^[^ ^5^, ^19^ ^]^ Finally, FemXpress includes a module to calculate the expression dosage ratio of the X chromosome to autosomes. Instead of using complex normalization methods, we calculated the ratio of sequencing reads that mapped to the X chromosome and autosomes directly. We observed consistent patterns in expression ratios among different cell types in the same sample (Figure 3h; Figure S3d,e, Supporting Information). Interestingly, the choroid plexus epithelium and ependymal cells exhibited lower levels of X chromosome‐derived gene expression (Figure 3h). This trend was consistently observed in additional publicly available mouse brain datasets (Figure 3i).

In summary, by analyzing both simulated and real scRNA‐Seq data, FemXpress exhibited several reliable capacities, including distinguishing parental sources of XCI, identifying XCI escapees, and analyzing the X chromosome expression dosage among different cell types in female single cells. These findings indicate that FemXpress is a powerful tool for XCI‐related studies.

### 2.4 Deciphering XCI Heterogeneity in Monkey Multi‐Tissue Data

**Figure 4:**
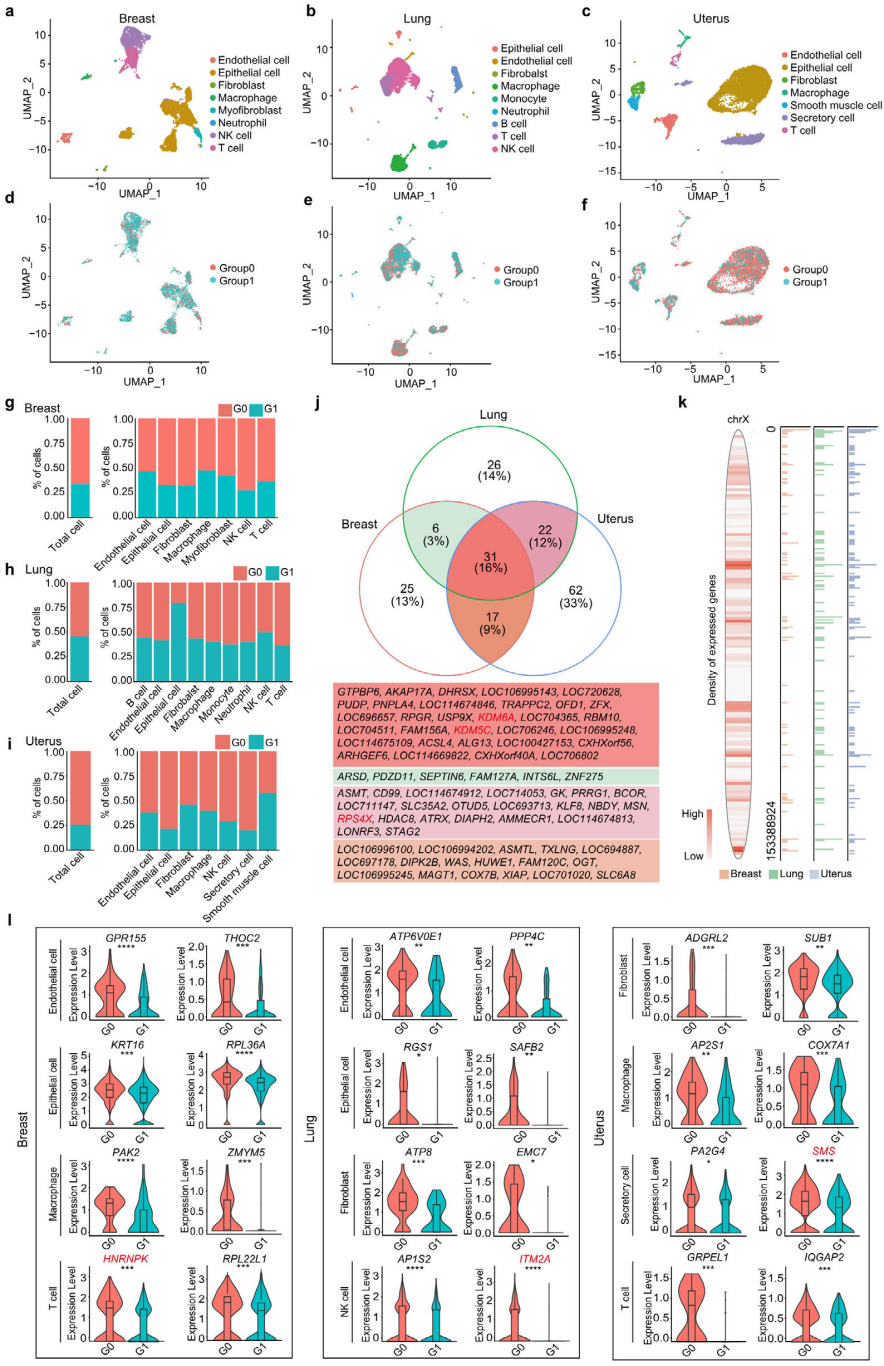
Utilization of FemXpress to analyze the X chromosome inactivation (XCI) pattern in the cynomolgus monkey cross‐tissue data. a–c) UMAP embedding of breast (a), lung (b), and uterus (c) in the cynomolgus monkey data, with each cell color‐coded by cluster identity andannotated by cell type. d–f) UMAP embedding of breast (d), lung (e), and uterus (f) in the cynomolgus monkey data, with cells color‐coded based on sample origin. g–i), Stacked histogram representing the distribution of cell XCI origins for each cell type across breast (g), lung (h), and uterus (i), as predicted by FemXpress. j) Venn plot showing the overlap of escapees in the three organs inferred by FemXpress. k) Density distribution of expressed genes in the three organs in the X chromosome (left). Density distribution of potential XCI‐escaping genes in the three organs along the X chromosome as inferred by FemXpress (right). l) Violin plots depicting the expression levels of selected DEGs between cells with varying parental XCI origins within specified cell types across each organ.

Building on the performance evaluation of FemXpress, we next explored the parental origin differences of XCI across organs by leveraging scRNA‐Seq data from multiple tissues of the same cynomolgus monkey individual to uncover the heterogeneity of XCI. After quality control, between 5,718 and 10,536 cells from the mammary gland, lung, and uterus were retained for XCI analysis (**Figure** **4**a–c).^[^ ^20^ ^]^ The FemXpress results revealed significant differences in the parental origin proportions of XCI across the three organs. Monkey lung exhibited nearly equal contributions from both parental origins, whereas the mammary gland and uterus showed a clear bias toward one parental origin, respectively. Furthermore, significant differences in parental origin proportions were also observed among different cell types within each organ (Figure 4d–i). This finding highlights the substantial heterogeneity of XCI across organs.

Subsequently, we identified 79–132 potential XCI‐escaping genes in each organ by leveraging the different allele bases between the maternal and paternal X chromosomes using FemXpress. Among these, a total of 31 potential escapees were shared by the three organs, 11 of which have been previously reported as XCI escapees in humans (Figure 4j). Further analysis of the distribution of these genes along the X chromosome revealed certain regions of enrichment for escape genes (Figure 4k).

Finally, we used FemXpress to identify genes that were differentially expressed between cells with maternal and paternal X chromosome inactivation within identical cell types. After filtering, a number of DEGs were identified across the various cell types in the three organs (Figure 4l). Interestingly, these differentially expressed genes included not only autosomal genes but also X‐linked genes. For instance, *ITM2A* and *SMS*, both located on the X chromosome and previously reported as escape genes,^[^ ^21^ ^]^ exhibited differential expression based on parental origins, suggesting that parental‐origin‐specific expression may serve as an additional mechanism for regulating gene dosage. Moreover, *HNRNPK*, an autosomal gene encoding a pre‐mRNA binding protein^[^ ^22^ ^]^, also showed differential expression in T cells of the breast, potentially contributing to the regulation of RNA heterogeneity among cells (Figure 4l).

In addition, we expanded our analysis to additional datasets. Specifically, we analyzed single‐cell data from female fetal embryos at four weeks postconception (PCW4) from different individuals at the same developmental stage to investigate inter‐sample heterogeneity in XCI and differences in XCI‐escaping genes.^[^ ^23^ ^]^ We also analyzed scRNA‐Seq data from female colon tumors of different patients^[^ ^24^ ^]^ and observed sample‐specific differences in XCI levels (Figures S4–S6, Supporting Information). These datasets further demonstrate the broad applicability of FemXpress in analyzing XCI in female single‐cell samples.

## 3 Discussion

In this study, we designed a novel tool, FemXpress, for XCI analysis. FemXpress can be used to divide cells into two groups based on the origin of the inactivated X chromosome and detect XCI‐escaping genes in single female cells by analyzing scRNA‐Seq data. The scRNA‐Seq data were sufficient to perform the analysis without any additional genomic information. Notably, FemXpress can identify the DEGs of each cluster divided by the different parental origins of XCI. To the best of our knowledge, FemXpress is a powerful tool developed to classify cells into two groups based on the origin of the inactivated X chromosome at the single‐cell level and further categorize cells with the same expression pattern into sub‐populations based on XCI origin.

FemXpress has applications in both developmental and clinical studies. For example, monozygotic twins can exhibit phenotypic differences. However, the underlying regulatory mechanisms have not yet been fully elucidated.^[^ ^25^ ^]^ Studies have reported discordant XCI escape and XCI heterogeneity in monozygotic twins.^[^ ^26^ ^]^ FemXpress is a valuable tool for supporting the investigation of phenotypic differences from the perspective of XCI heterogeneity. Additionally, the dysregulation of XCI, including disordered inactivation escaping and skewing, has been linked to disease onset. Studies have shown that over 41% of females above the age of 65 years experienced an XCI skew, which has been closely associated with tumorigenesis and immune system diseases.^[^ ^7^, ^8^ ^]^ This indicates the underlying application of FemXpress in the identification of diagnostic markers or therapeutic targets by analyzing XCI skew and escapees at the single‐cell resolution.

Scphaser is a tool capable of performing chromosome phasing after modifications to its R code. However, it is better suited for high‐depth sequencing data, such as smart‐Seq, and does not perform well on sparse datasets such as those generated by 10x Genomics. Vireo, another phasing tool based on variational Bayesian inference of latent variable distributions, performs better in handling such sparse data. Nevertheless, because it relies on approximate inference, its phasing accuracy is generally lower than that achieved by FemXpress. scLinaX, developed for analyzing XCI heterogeneity at the single‐cell level, is able to quantify gene‐specific escape across different cell populations. However, it does not support the classification of cells based on the parental origin of the inactivated X chromosome. FemXpress, by contrast, leverages the unique transcriptional characteristics of the X chromosome in normal somatic cells‐specifically, the fact that only one allele of the X chromosome is generally expressed, aside from a few escape genes. This allows FemXpress to effectively phase the X chromosome using single‐cell transcriptomic data. In the future, integrating the strengths of these tools with FemXpress for XCI inference could further enhance model performance.

Although FemXpress does not require any additional genomic information, it does require sufficient SNP differences between the parental X chromosomes to precisely divide cells into two groups based on the origin of the inactivated X chromosome. In the absence of SNP information, FemXpress is currently unable to determine the parental origin of the inactivated X chromosome. However, given the rapid advances in artificial intelligence (AI) technology, AI‐based models may offer promising solutions to infer the parental origin of XCI based solely on gene expression patterns.^[^ ^27^ ^]^ A current limitation of FemXpress is its reliance on a sufficient number of high‐quality SNPs. In the future, the integration of algorithms such as imputation and bootstrapping could help recover low‐signal SNPs, enabling FemXpress to function effectively even with sparse SNP data. This would significantly broaden its applicability across a wider range of biological and technical contexts. In summary, the advent of FemXpress opens a new avenue for analyzing cellular functions and phenotypic differences in female cells attributable to variations in the source of XCI, providing novel perspectives for the study and elucidation of XCI.

## 4 Experimental Section

### Animals

The C57BL/6NJ and PWk/PHJ mice were purchased from Beijing Vital River Laboratory Animal Technology Co., Ltd. All animal studies were performed in accordance with the Guidelines for the Use of Animals in Research issued by the Institute of Zoology of the Chinese Academy of Sciences.

### Preparation of Single‐Cell Suspensions

The tissues were transferred into new EP tubes containing precooled LB Buffer and cut into pieces. After lysing on the ice for 5–10 min, the solution was passed through a 40‐µm filter into a new EP tube and then centrifuged at 500 × *g* for 5 min at 4 °C. The supernatant was removed carefully and 300 µL of LB Buffer was added to resuspend the pellet. Then, 300 µL of RB Buffer was added and the solution was mixed gently. Next, 600 µL of PB1 Buffer was added from the bottom of the tube with pipette tip slowly, followed by the addition of 600 µL of PB2 Buffer from the bottom of the tube slowly. The solution was then centrifuged at 4000 × *g* for 20 min at 4 °C. The resulting nuclei layer between PB1 and PB2 was transferred into a new EP tube, to which 1 mL of RB Buffer was added. Then, the pellet was resuspended gently and the solution was passed through a 40‐µm filter before centrifuging at 500 × *g* for 5 min at 4 °C. Lastly, the supernatant was carefully removed from the tube and 100 µL of EB Buffer was added to resuspend the nuclei. The resulting single‐nucleus suspension was used for further analysis.

### Single‐Cell RNA Sequencing

Using the Chromium Single Cell 3′ Library and Gel Bead Kit v3.1 and the Chromium Single Cell B Chip Kit, the cell suspension (300–600 live cells per microliter, as determined by Countstar) was loaded onto the Chromium Single Cell Controller to generate single‐cell gel beads in emulsion, following the manufacturer’s protocol. Briefly, single cells were suspended in PBS containing 0.04% BSA. ≈6000 cells were added to each channel, and the target cell to be recovered was estimated to contain ≈3000 cells. The captured cells were lysed and the released RNA was barcoded through reverse transcription in individual GEMs. Reverse transcription was performed on a S1000TM Touch Thermal Cycler (BioRad) at 53 °C for 45 min, followed by 85 °C for 5 min, and held at 4 °C. cDNA was generated and amplified, and its quality was assessed using an Agilent 4200 (Capital Bio‐Technology, Beijing). Single‐cell RNA‐Seq libraries were constructed according to the manufacturer's instructions using the Single Cell 3 Library and Gel Bead Kit V3.1. Finally, the libraries were sequenced using an Illumina NovaSeq 6000 sequencer with a sequencing depth of at least 100,000 reads per cell using a paired‐end 150 bp (PE150) reading strategy.

### Single‐Cell RNA‐Seq Analysis

The raw data was aligned to the reference genome mm10 using Cell Ranger in include‐intron mode, from which a barcode‐count matrix was generated. Seurat was used for downstream analysis. Cells with fewer than 200 unique genes, greater than 30% ribosomal counts, greater than 10% hemoglobin counts, and all nuclei with greater than 30% mitochondrial counts were excluded from subsequent analyses. An average number of 1909 expressed genes per cell and an average number of 3088 counts per cell were detected. Data were scaled and normalized, and the default 2000 highly variable genes were selected using Find Variable Features for future analyses. The first 30 dimensions were selected for principal component analysis (PCA). Dimensionality reduction and visualization were performed using RunUMAP. FindAllMarkers was used to identify the marker genes in each cluster. Potential doublets were identified using DoubletFinder, after which the identified potential doublets were removed and the remaining cells were subjected to the same analysis workflow (scaling, normalization, dimensionality reduction, and clustering). Different classical cell‐type markers were used to annotate the different cell types. DEGs between different cell types or different groups of the same cell type were identified using FindAllMarkers with the following parameters (min pct = 0.1, logfc threshold = 0.25).

### Whole‐Genome Sequencing (WGS) Analysis

Raw data were aligned to the reference genome mm10 using bowtie2, and base count was used to count the bases at each location on the chromosome. Mouse strain‐specific SNP positions could be distinguished, which represented the gold standard for measuring the source of XCI in each cell.

### Data Preprocessing of the Final SNP‐Barcode Matrix

Freebayes was used to search for mutations in BAM files containing high‐quality barcodes after control quality, where SNP variants with genotype 0/1 were retained for tighter filtering. Next, the COUNT and RATIO values were calculated for all barcodes in each sample. To this end, the barcodes’ count for each SNP position was recorded. The next largest cumulative number of four bases in all cells at each SNP position represents the COUNT value, while RATIO was the ratio of the barcodes’ number of the largest two bases. For example, if the cumulative number of ACGT bases in a barcode was 100, 30, 0, and 0, then the RATIO was 3.3333, while the cumulative number of ACGT bases in a barcode was 5, 50, 0, and 0, then the RATIO was 0.1. The filtering criteria were as follows: (1) the 21‐bp base sequence, which comprises the upstream and downstream 10‐bp centered on each SNP position, contains only two bases (A, C, G, or T); (2) the 21‐bp sequence contains the same base occurring five times or more in a row (e.g., GCATTCAAAAAAAGTCATGCG; in this 21‐bp sequence, “A” appears six times in a row); (3) the 21‐bp sequence contains the same base 10 times or more (e.g., GGCGCTCGAGGGTCTCGGGCT; in this 21‐bp sequence, “G” appears 10 times); (4) COUNT > 2; (5) 0.1 < RATIO < 0.9. After filtering based on the above five criteria, all the remaining high‐quality SNP positions were used to construct the SNP‐barcode matrix as an input to FemXpress.

### Matrix Processing and Allele Determination (Part1 of FemXpress Workflow)

FemXpress starts from an SNP‐barcode matrix, whose rows were cell barcodes and whose columns were SNP positions on the X chromosome. The matrix was comprised of the number of reads supporting the position presenting A, C, G, or T separated by semicolons. FemXpress scans and reads the matrix, and determines the actual base of each position by taking the base with the maximum number of supporting reads. Only two alleles (maternal and paternal) were assumed at each position in the entire cell population. Thus, the identified alleles, other than those with the most supporting cells, were deleted and were not considered in the following steps.

### Escaping Gene Inference (part2 of FemXpress Workflow)

For each cell group, every SNP position in the SNP‐barcode matrix was examined to identify those for which a substantial fraction of cells supported one allele and another substantial fraction supported the alternative allele. After testing various thresholds, the permissible range of allele support to was set to 30–70%, and required at least five supporting cells per allele was required. SNP positions meeting these criteria in both groups were inferred as escaping positions. Any gene containing at least one escaping position was subsequently classified as an XCI‐escaping gene.

### Simulated Data Generation

We downloaded 3‐year, 9‐month, 15‐day UK female heart tissue single‐nucleus RNA‐Seq data released in the NCBI SRA under accession SRR19266894. The sequenced reads were aligned to the GRCh38 build of the human reference genome using Cell Ranger. To simulate a set of single‐cell RNA‐Seq data of a female and use the female parents’ genotypes as the gold standard for FemXpress X chromosome origin inference, recalibrated variants of chromosome X from the 1000 Genomes Family Trio of a southern Han Chinese population of East Asian ancestry were downloaded under accessions HG00403 (father), HG00404 (mother), and HG00405 (female child) from The International Genome Sample Resource website. Using the Python Pysam module, the reads of the BAM file of the aligned SRR19266894 reads were modified so that all mismatched bases were changed to the reference allele. All the read positions were identified where the origin of the two HG00405 alleles could be determined as one allele inherited from HG00403 and the other allele from HG00404, and those positions on the reads were modified to one of the HG00405 alleles for each cell in the BAM file. The confirmed escaping gene list was extracted from the reference,^[^ ^28^ ^]^ and reads positions overlapping with the genes in the escaping list were modified to randomly select the child's alleles when simulating XCI‐escaping genes. A gradient of random sequencing errors was introduced by random modifications to each sequencing read at specific rates (0.01%, 0.02%, 0.05%, 0.1%, 0.2%, 0.5%, 1%, 2%, and 5%). The modified reads for each setting of error rate, cell barcodes, and UMIs were extracted from the BAM file to produce a pair of FASTQ files.

### Linkage identification (part1 of Matrix processing and allele determination)

For linkage identification, It was assumed that the input matrix had *p* SNP positions, and the genotype vector was {*G*_1_, *G*_2_, ⋅⋅⋅,  *G_p_*}. We denoted $G_{i}=A_{i}^{1}|A_{i}^{2}$, where · | · indicates the phased genotype. The genotype matrix can be represented as:

(1) $$G=\left[\begin{array}{ccc} b_{11}^{1}|b_{11}^{2} & \text{…} & b_{1c}^{1}|b_{1c}^{2}\\ \vdots & & \vdots \\ b_{p1}^{1}|b_{p1}^{2} & \text{…} & b_{pc}^{1}|b_{pc}^{2} \end{array}\right]$$

where *c* denotes the number of cells. $b_{ij}^{1}$ and $b_{ij}^{2}$ denote the bases of the $A_{i}^{1}$ and $A_{i}^{2}$ alleles, respectively, in cell *j*, respectively. For each pair of genotypes, *G_s_*, *G_l_* that present in at least one cell in the cell population, It is calculated

(2) $$\begin{array}{c} p\left(A_{s}^{1}\left|A_{s}^{2},A_{l}^{1}\right|A_{l}^{2}\right)=\sum_{j}\frac{c\left(b_{sj}^{1},b_{lj}^{1}\right)+c\left(b_{sj}^{2},b_{lj}^{2}\right)}{c\left(b_{sj}^{1},b_{lj}^{1}\right)+c\left(b_{sj}^{2},b_{lj}^{2}\right)+c\left(b_{sj}^{1},b_{lj}^{2}\right)+c\left(b_{sj}^{2},b_{lj}^{1}\right)} \end{array}$$

and

(3) $$\begin{array}{c} p\left(A_{s}^{1}\left|A_{s}^{2},A_{l}^{2}\right|A_{l}^{1}\right)=\sum_{j}\frac{c\left(b_{sj}^{1},b_{lj}^{2}\right)+c\left(b_{sj}^{2},b_{lj}^{1}\right)}{c\left(b_{sj}^{1},b_{lj}^{1}\right)+c\left(b_{sj}^{2},b_{lj}^{2}\right)+c\left(b_{sj}^{1},b_{lj}^{2}\right)+c\left(b_{sj}^{2},b_{lj}^{1}\right)} \end{array}$$

where $c(b_{sj}^{1},b_{lj}^{1})$ denotes the number of co‐occurrences of $b_{sj}^{1}$ and $b_{lj}^{1}$ in cell *j*, $p(A_{s}^{1}\left|A_{s}^{2},A_{l}^{1}\right|A_{l}^{2})$ denotes the probability of seeing $b_{sj}^{1},b_{lj}^{1}$ or $b_{sj}^{2},b_{lj}^{2}$ in the same cell in the cell population, and $p(A_{s}^{1}\left|A_{s}^{2},A_{l}^{2}\right|A_{l}^{1})$ denotes the probability of seeing $b_{sj}^{1},b_{lj}^{2}$ or $b_{sj}^{2},b_{lj}^{1}$ in the same cell in the cell population. $A_{s}^{1},A_{l}^{1}$ and $A_{s}^{2},A_{l}^{2}$ were used as the linkages between positions *s* and *l*. If $p(A_{s}^{1}\left|A_{s}^{2},A_{l}^{1}\right|A_{l}^{2})$ was greater than a certain threshold, the default was 0.8. Similarly, if $p(A_{s}^{1}\left|A_{s}^{2},A_{l}^{2}\right|A_{l}^{1})$ was greater than the threshold, $A_{s}^{1},A_{l}^{2}$ and $A_{s}^{2},A_{l}^{1}$ were used as the linkages between positions *s* and *l*. After iterating through all pairs of positions that may have a linkage, a set of linkages was obtained from the matrix.

### Haplotype Phasing (part2 of Matrix Processing and Allele Determination)

We constructed both haplotypes (maternal and paternal) by connecting linkages from the linkage set obtained in the previous step. The rule of connection was based on the fact that if $A_{s}^{1},A_{l}^{1}$ were linkages and $A_{l}^{1},A_{m}^{1}$ were linkages, $A_{s}^{1},A_{l}^{1},A_{m}^{1}$, were connected and used them as the scaffold of one of the haplotypes. The other haplotype scaffolds were $A_{s}^{2},A_{l}^{2},A_{m}^{2}$ correspondingly. A set of linkages was used, and the scaffolds greedily and iteratively extended to construct the fragments of the two haplotypes required. Fragments whose linkage numbers exceeded the score threshold (default = 5) were collected as the fragment set to merge in the next step.

### Fragment merge and cell classification (part3 of Matrix processing and allele determination)

For a qualified fragment $A_{s}^{1},A_{l}^{1},A_{m}^{1},\text{…}$ and cell *c*, if the genotype of *c* was $A_{s}^{1}$ at position *s*, then *c* had one vote from haplotype 1. Otherwise, if the genotype of *c* was $A_{s}^{2}$ at position *s*, *c* would have one vote from haplotype 2. Cell *c* was conducted based on each position on the fragment. If, for cell *c*, the total number of votes from haplotype 1 was greater than the total number of votes from haplotype 2, *c* was taken as a haplotype 1 cell, and vice versa. Thus, fragment $A_{s}^{1},A_{l}^{1},A_{m}^{1},\text{…}$ divided a fraction of all the cells into two classes. By repeating these steps for all the constructed fragments and merging pairs of fragments whose cell divisions had an overlap exceeding a threshold (50% by default) between fragments, the fragments of the haplotypes were merged.. The cells were then classified by voting in the same manner as the two merged haplotypes to obtain the final cell classification result.

### Iterating to Find the Best Parameters (part4 of Matrix Processing and Allele Determination)

Each SNP position may have different reads coverage, and low reads coverage may indicate a sequencing error. Therefore, the last three steps were reran 10 times with different SNP read coverage to determine the most credible cutoff. This result was considered to be the output of the algorithm.

### Simulated Data Processing

The FASTQ files of the modified reads were aligned to the GRCh38 build of the human reference genome using Cell Ranger. Variants were detected using Freebayes, with the parameter C set to 1. Variant calling was refined using an in‐house script that modified multiple unseparated single nucleotide variants (SNV) to separate the SNVs (e.g., ref: ATCGT, alt: CTAGC was separated into ref: A, alt: C, ref: C, alt: A and ref: T, alt: C). The read numbers supporting A, C, G, or T at each genomic position in each cell were counted, and the results were transformed into an SNP‐barcode matrix whose rows represented cells and columns represented genomic positions on chromosome X. Simulated samples with the error rate gradient were processed using FemXpress, Vireo and scphaser, and the output clustering results were evaluated using in‐house scripts against the gold standard of parent (HG00403 and HG00404) genotypes.

FemXpress, along with scripts for reproducing key analysis results, is available in open source at https://github.com/aprilW0829/FemXpress.

## Conflict of Interest

The authors declare no conflict of interest.

## Author Contributions

X.W., Y.M., F.L., and W.C. contributed equally to this work. G.F, Q.Z., P.W. W.L. and Y.M. conceived and designed the study. G.F. supervised the study and data analysis. X.W., Y.M., F.L. and W.C. developed the method, wrote the original code, and analyzed the data. T.P. S.M., Q.S. and C.L. prepared the samples for sequencing. G.F, Q.Z., Y.M., S.W., Y.W. and Y.Z. wrote the manuscript with input from all authors. All authors approved the final version submitted.

## Supporting information



Supporting Information

## Data Availability

The original sequencing data of mouse brain in this study have been deposited in the Genome Sequence Archive in the National Genomics Data Center, Beijing Institute of Genomics (China National Center for Bioinformation) of the Chinese Academy of Sciences, with accession number CRA015497.
