# Supplementary material for: FemXpress: Systematic Analysis of X Chromosome Inactivation Heterogeneity in Female Single‐Cell RNA‐Seq Samples
Source: Adv Sci (Weinh). 2025 Jun 29;12(35):e04754. doi: 10.1002/advs.202504754 (PMC12463006; doi:10.1002/advs.202504754)
Supplement: Supplementary file 1 — Supporting Information [file ADVS-12-e04754-s001.docx]

**Figure S1**

**
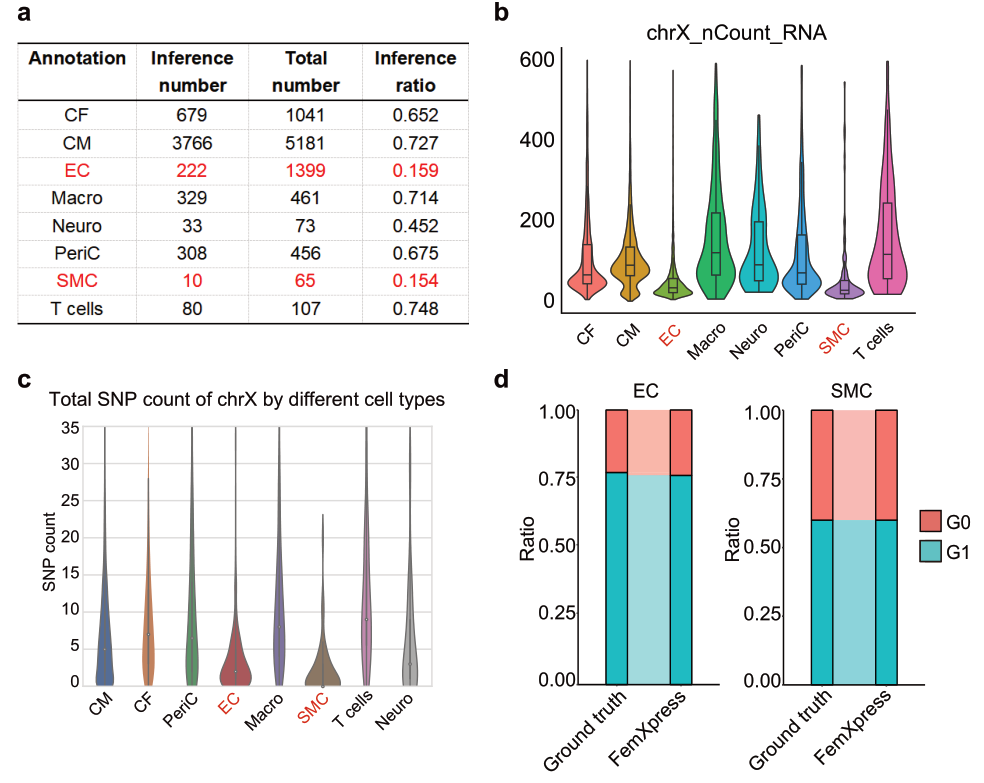
**

**Figure S1.** Factors influencing FemXpress inference success in the simulated data. a) The table showing the number of cells, the number of cells inferred by FemXpress and the proportion of cells inferred by FemXpress for each cell type in the simulated data (CM, cardiomyocyte; CF, cardiac fibroblast; EC, endothelial cell; EpiL, epithelial-like; PeriC, pericyte; Neuro, neuron; SMC, smooth muscle cell). b) Violin plot showing the distribution of total number of reads on the X chromosome in each cell type in the simulated data. c) Violin plot showing the distribution of X-chromosome SNP counts in the simulated data. d) Bar plot showing the proportion of correctly inferred cells among those that were successfully inferred by FemXpress in each cell type.

**Figure S2**


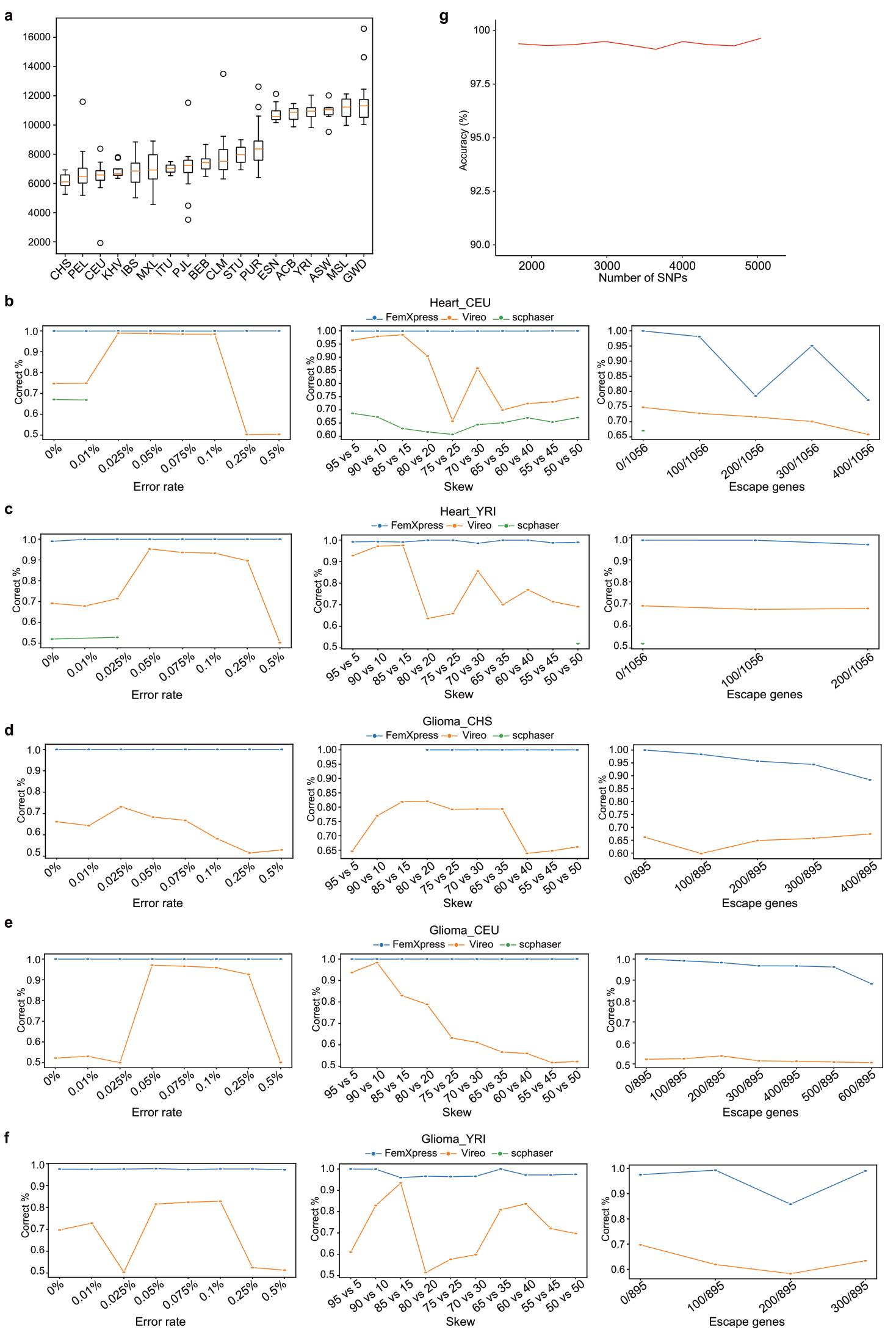


**Figure S2.** Benchmark of FemXpress clustering function with simulated data across different populations and scRNA-Seq datasets. a) Population-stratified analysis of 289 female-child family trios from the 1000 Genomes Project, illustrating the number of child SNPs for which the parental origin could be determined. b-f) Benchmark comparison of FemXpress, Vireo, and scphaser clustering functions using simulated data derived from two scRNA-Seq datasets (human heart tissue and human glioma) and three human populations (CHS, CEU, and YRI). Due to the high memory requirements of scphaser, full results were not obtained for all simulations. For the Heart_CHS results and a detailed explanation of the simulated data, refer to Figure 2b-d and the corresponding figure legends.

**Figure S3**

**
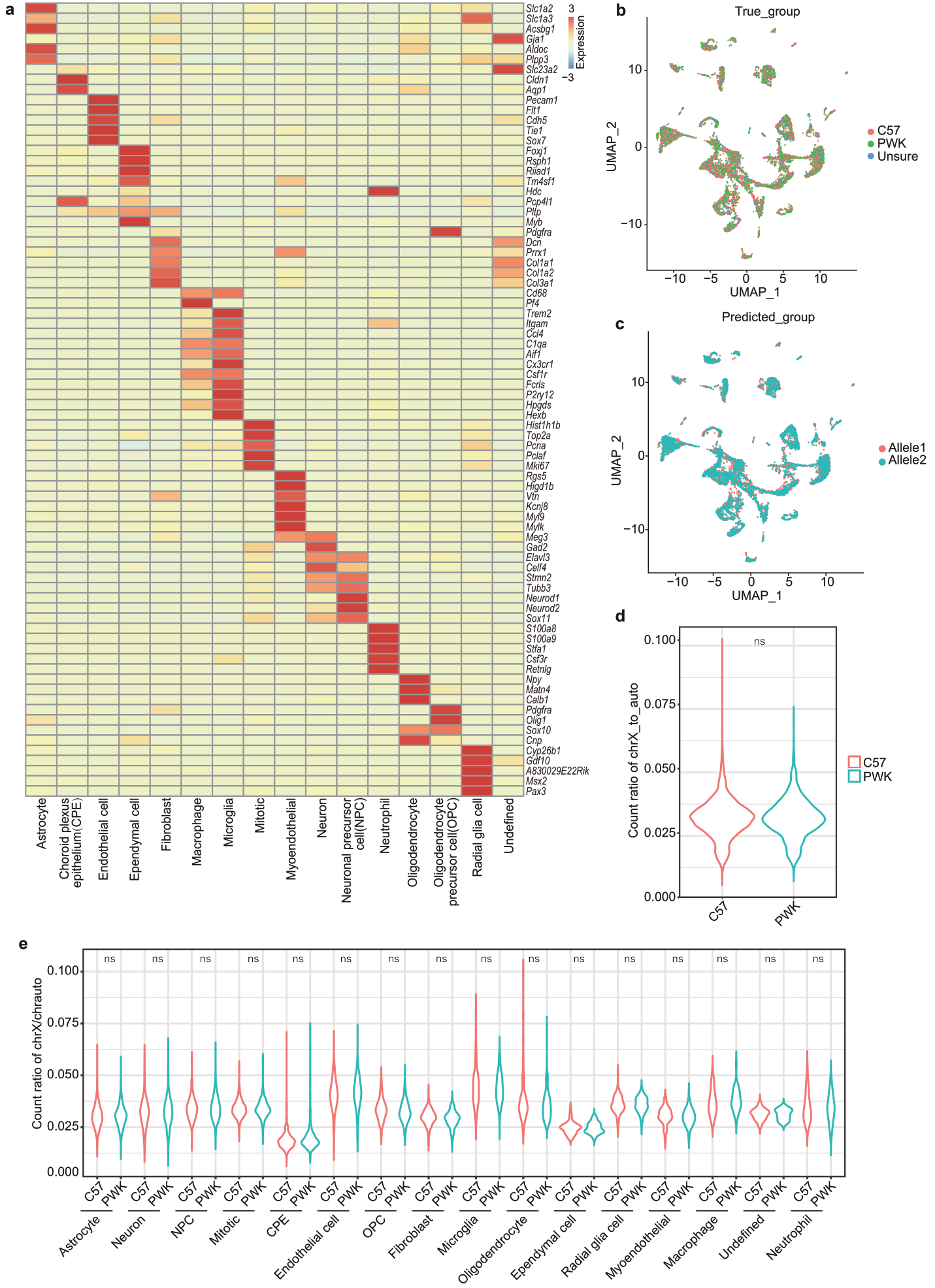
**

**Figure S3.** Single-cell transcriptomics and FemXpress analysis of brain from crosses between the C57BL/6NJ and PWk/PHJ strains. a) Heatmap showing marker genes of different cell types from the mouse brain. b) The UMAP embedding shows mouse brain scRNA-Seq data with cells color-coded by XCI origin: the validated ground truth. c) The UMAP embedding shows mouse brain scRNA-Seq data with cells color-coded by XCI origin: FemXpress’s classification. d) Violin plots showing the X-to-autosome read count ratios across two FemXpress’s classification of all cells in mouse brain tissue. e) Violin plots showing the X-to-autosome read count ratios across two FemXpress’s classification of different cell types in mouse brain tissue.

**Figure S4**


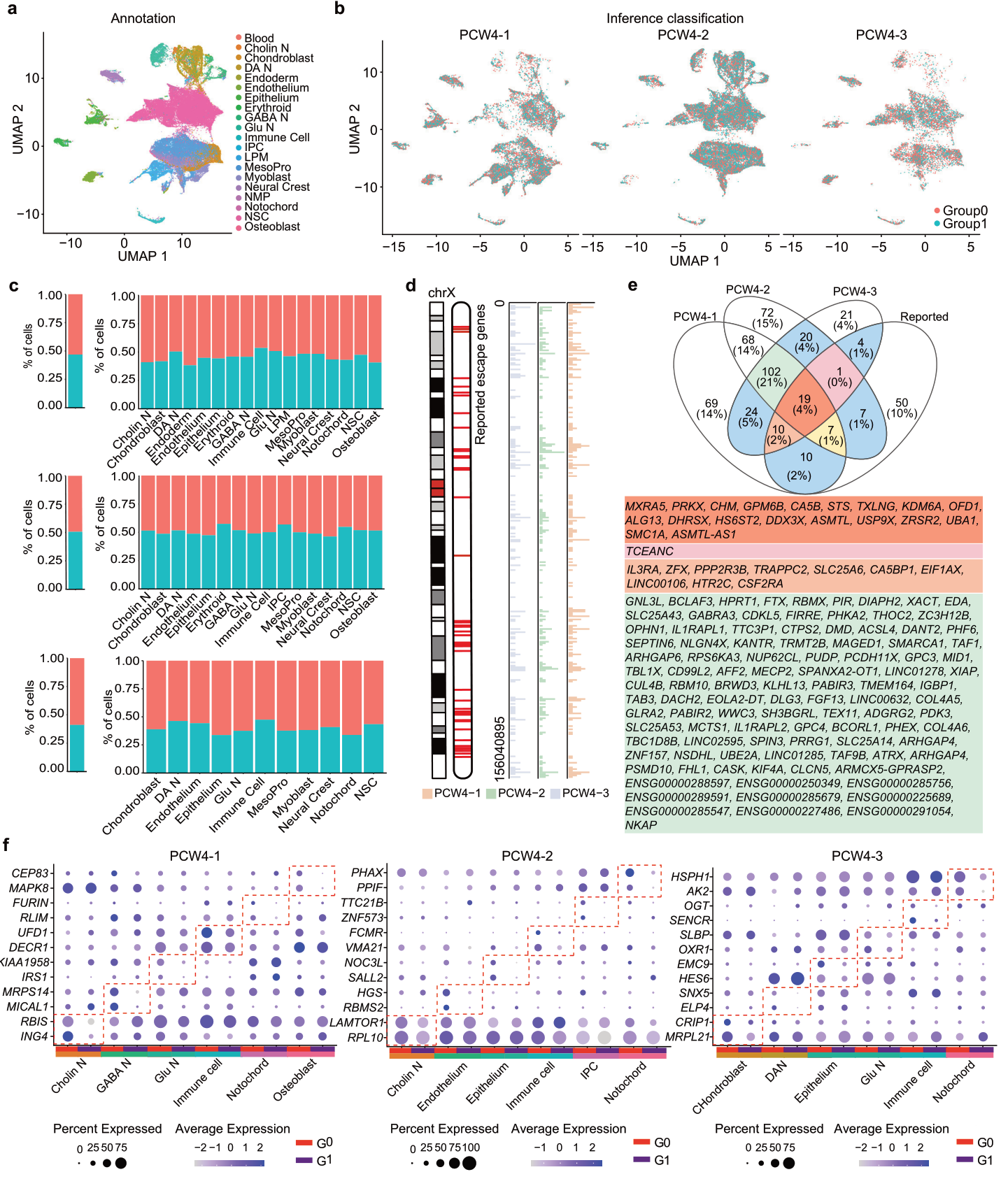


**Figure S4.** Utilization of FemXpress to analyze the X chromosome inactivation (XCI) pattern in female embryonic samples. a) UMAP embedding of human embryonic scRNA-Seq data, with each cell color-coded according to cluster identity and annotations denoting the respective cell types (Cholin N, cholinergic neurons; DA, dopaminergic neurons; GABA N, GABAergic neurons; Glu N, glutamatergic neurons; IPC, intermediate progenitor cells; LPM, lateral plate mesoderm; MesoPro, proliferating mesoderm; NMPs, neuromesodermal progenitors; NC, neural crest cells; NSCs, neural stem cells). b) UMAP embedding of human embryonic scRNA-Seq data, with cells color-coded based on sample origin. c) Stacked histogram showing the distribution of cell XCI origins for each cell type across three samples, as inferred by FemXpress. d) Density distribution of potential XCI-escaping genes in human tissue along the X chromosome as inferred by FemXpress (left). Venn diagram depicting the overlap of escaping genes among three samples and the reported gene list ^[[28](#_ENREF_37)]^. e) Venn plot showing potential escapees in the three samples inferred by FemXpress and reported human escapees. f) Dot plots depicting the expression levels of selected DEGs between cells with varying parental XCI origins within specified cell types across each sample.

**Figure S5**


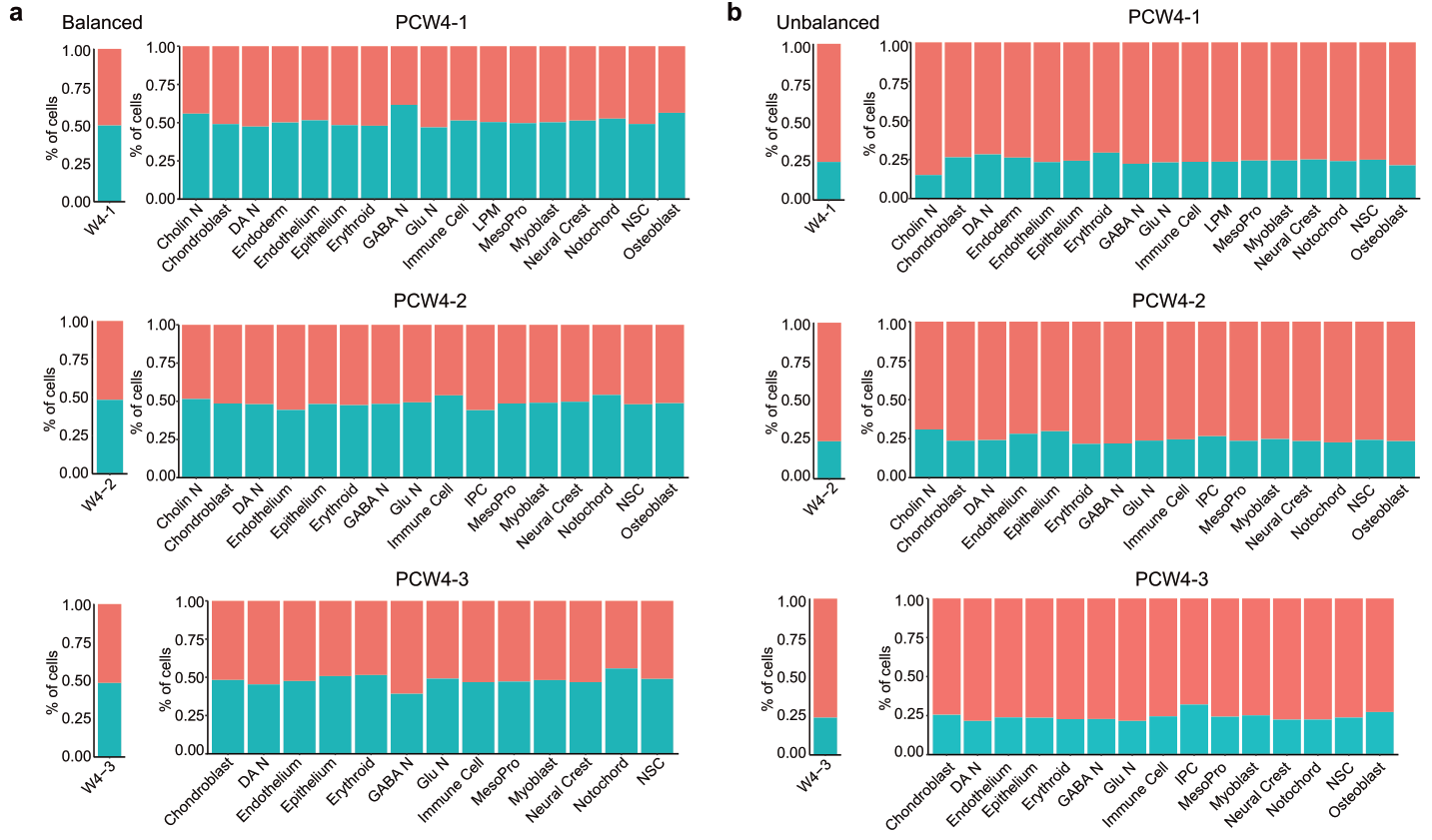


**Figure S5.** FemXpress inference results in three human embryo samples. a) Bar plots depicting simulated data generated using PolyP, combined with unaffected scRNA-Seq expression data and SNP data from the 1000 Genomes family trio (HG00403, HG00404, and HG00405). The simulation assumes each cell's X chromosome has a 50% probability of being inherited from the father and 50% from the mother. These plots are designed to evaluate the performance of FemXpress on human colon scRNA-Seq data when the paternal-to-maternal X chromosome inheritance ratio is balanced (deviating from the expected 50:50 distribution). b) Bar plots depicting simulated data generated using PolyP, combined with unaffected scRNA-Seq expression data and SNP data from the 1000 Genomes family trio (HG00403, HG00404, and HG00405). The simulation assumes each cell's X chromosome has a 75% probability of being inherited from the father and 25% from the mother. These plots are designed to evaluate the performance of FemXpress on human colon scRNA-Seq data when the paternal-to-maternal X chromosome inheritance ratio is unbalanced (deviating from the expected 50:50 distribution).

**Figure S6**


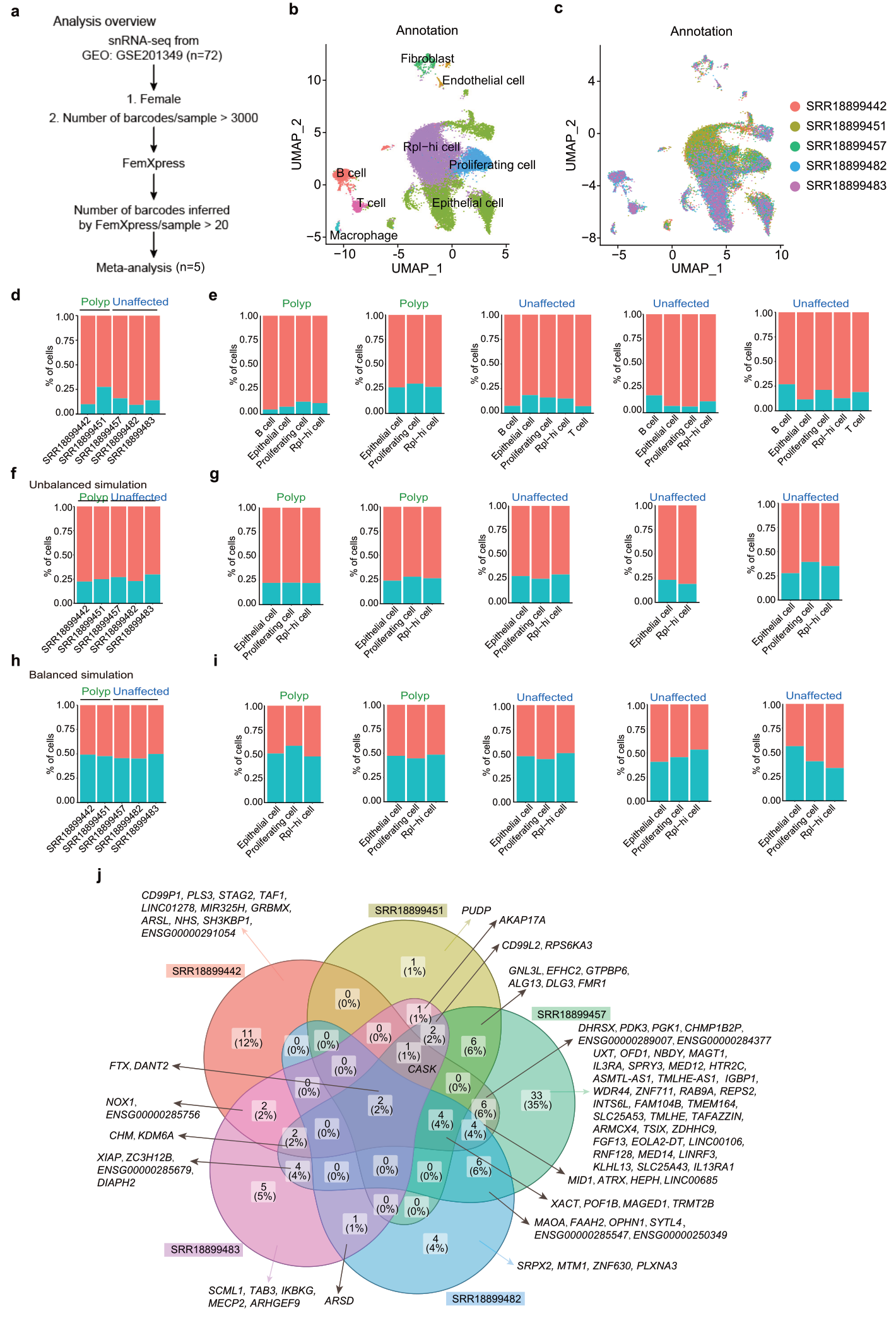


**Figure S6.** FemXpress inference results in human colon samples. a) Workflow used to select high-quality colon tumor samples from female patients. b) UMAP embedding of the filtered colon tumor dataset. Each cell is color-coded by cluster identity and annotated according to its cell type (Rpl-hi cell: Cells with high expression of ribosomal genes). c) UMAP embedding of the same dataset, with cells color-coded by their respective sample origins. d) Bar plot showing the overall proportions of cells classified into each XCI origin (e.g., maternal or paternal) as inferred by FemXpress. e) Bar plots illustrating the proportions of cells, categorized by cell type, that were assigned to each XCI origin in individual female samples. f) Bar plot depicting simulated data generated using PolyP, combined with unaffected scRNA-Seq expression data and SNP data from the 1000 Genomes family trio (HG00403, HG00404, and HG00405). The simulation assumes each cell's X chromosome has a 75% probability of being inherited from the father and 25% from the mother. These plots are designed to evaluate the performance of FemXpress on human colon scRNA-Seq data when the paternal-to-maternal X chromosome inheritance ratio is unbalanced (deviating from the expected 50:50 distribution). g) Bar plots illustrating the distribution of inferred X-chromosome inactivation (XCI) origins by cell type for each sample in the simulated data described in f. h) Bar plot depicting simulated data generated using PolyP, combined with unaffected scRNA-Seq expression data and SNP data from the 1000 Genomes family trio (HG00403, HG00404, and HG00405). The simulation assumes each cell's X chromosome has a 50% probability of being inherited from the father and 50% from the mother. These plots are designed to evaluate the performance of FemXpress on human colon scRNA-Seq data when the paternal-to-maternal X chromosome inheritance ratio is balanced (aligning with the expected 50:50 distribution). i) Bar plots illustrating the distribution of inferred X-chromosome inactivation (XCI) origins by cell type for each sample in the simulated data described in f. J) Escapees identified by FemXpress across three different female colon tumor samples.
